# Turning to the dark side: LED light at night alters the activity and species composition of a foraging bat assemblage in the northeastern United States

**DOI:** 10.1002/ece3.7466

**Published:** 2021-03-27

**Authors:** Chad L. Seewagen, Amanda M. Adams

**Affiliations:** ^1^ Great Hollow Nature Preserve & Ecological Research Center New Fairfield CT USA; ^2^ Department of Natural Resources and the Environment University of Connecticut Storrs CT USA; ^3^ Wildlife and Fisheries Conservation Center University of Connecticut Storrs CT USA; ^4^ Bat Conservation International Austin TX USA; ^5^ Department of Biology Texas A&M University College Station TX USA

**Keywords:** artificial light at night, community composition, light‐emitting diode, *Myotis lucifugus*

## Abstract

Artificial light at night (ALAN) is a rapidly intensifying form of environmental degradation that can impact wildlife by altering light‐mediated physiological processes that control a broad range of behaviors. Although nocturnal animals are most vulnerable, ALAN's effects on North American bats have been surprisingly understudied. Most of what is known is based on decades‐old observations of bats around street lights with traditional lighting technologies that have been increasingly replaced by energy‐efficient broad‐spectrum lighting, rendering our understanding of the contemporary effects of ALAN on North American bats even less complete. We experimentally tested the effects of broad‐spectrum ALAN on presence/absence, foraging activity, and species composition in a Connecticut, USA bat community by illuminating foraging habitat with light‐emitting diode (LED) floodlights and comparing acoustic recordings between light and dark conditions. Lighting dramatically decreased presence and activity of little brown bats (*Myotis lucifugus*), which we detected on only 14% of light nights compared with 65% of dark (lights off) and 69% of control (lights removed) nights. Big brown bat (*Eptesicus fuscus*) activity on light nights averaged only half that of dark and control nights. Lighting did not affect presence/absence of silver‐haired bats (*Lasionycteris noctivagans*), but decreased their activity. There were no effects on eastern red bats (*Lasiurus borealis*) or hoary bats (*L. cinereus*), which have been described previously as light‐tolerant. Aversion to lighting by some species but not others caused a significant shift in community composition, thereby potentially altering competitive balances from natural conditions. Our results demonstrate that only a small degree of ALAN can represent a significant form of habitat degradation for some North American bats, including the endangered little brown bat. Research on the extent to which different lighting technologies, colors, and intensities affect these species is urgently needed and should be a priority in conservation planning for North America's bats.

## Graphical abstract

Experimental lighting of a wetland foraging habitat in Connecticut, USA reduced the presence and/or activity of big brown bats, little brown bats, and silver‐haired bats, while having no effect on the presence or activity of eastern red bats and hoary bats. This aversion to the lighting by some bat species but not others resulted in a significant change in community composition, thereby potentially altering competitive balances from natural conditions.

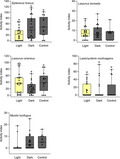

## 1 INTRODUCTION

Light pollution, also referred to as artificial light at night (ALAN), is a rapidly intensifying form of environmental degradation and a growing threat to biodiversity around the world (Gaston et al., 2015; Hölker et al., 2010; Koen et al., 2018). It can have myriad adverse effects on wildlife by altering circadian rhythms and other light‐mediated physiological processes (Beier, 2006; Gaston et al., 2017; Longcore & Rich, 2006). These imbalances can shift the timing of diel activities and cause wide‐ranging behavioral changes that affect space use, foraging, predator‐prey interactions, communication, migration, and reproduction (Gaston et al., 2014, 2017; Gauthreaux & Belser, 2006). Nocturnal animals, such as bats, are perhaps the most vulnerable to effects from light pollution because of its disruption of the dark conditions to which these species and their predators and prey have become adapted and specialized over many millennia (Beier, 2006).

ALAN has been found to be detrimental to some bat species while simultaneously appearing to benefit others. Those that appear to benefit are bats that forage in illuminated areas to take advantage of the high densities and weakened predator avoidance abilities of insects that are attracted to the light. Foraging activity and food intake rates of such species can be substantially greater in artificially illuminated areas than in dark areas (e.g., Blake et al., 1994; Cravens et al., 2018; Geggie & Fenton, 1985; Rydell, 1992). They tend to be “fast‐flying” species that are specialized at foraging over large, open spaces. In contrast, the bats that are relatively averse to foraging in areas with artificial lighting or otherwise adversely affected by lights are usually “slow‐flying” and clutter‐adapted species, commonly of the genus *Myotis* (McGuire & Fenton, 2010; Rowse et al., 2016; Rydell, 1992; Stone et al., 2009).

Most of what is known about the responses of bats to ALAN comes from research on European species. Mostly through field experiments, much has been learned in recent years about the effects of various lighting types, colors, and intensities on Europe's bats (e.g., Lewanzik & Voigt, 2017; Mathews et al., 2015; Stone et al., 2009; Voigt et al., 2020; Zeale et al., 2018). By comparison, the effects of lighting on bats in other parts of the world, including North America, remain poorly understood. Cravens and Boyles (2019) recently provided some of the first experimental information about the effects of ALAN on the foraging activity and energetics of free‐living North American bats by introducing light into previously dark woodland habitats in Missouri, USA. They too found some species to be attracted to the lighting and benefit energetically from the concentration of prey while others, including the three *Myotis* species in the community, generally avoided it. Otherwise, what is known about the effects of ALAN on North American bats is largely limited to decades‐old observations of bats around street lights that used traditional lighting technologies that are being increasingly replaced by energy‐efficient broad‐spectrum lighting, and landscape‐level associations of species with either heavily (e.g., urban) or minimally (e.g., rural) light‐polluted environments. This lack of information impedes science‐based management and the mitigation potentially needed to protect North American bats from both existing sources of ALAN and the rapid encroachment of new sources of ALAN into natural areas as development proceeds.

Here, we tested the effects of broad‐spectrum light‐emitting diode (LED) lighting on the foraging activity of a northeastern United States bat community to obtain some of the first experimental information about the sensitivity of these species to ALAN when it is introduced to a previously dark environment. We then asked whether species‐specific responses to ALAN significantly alter foraging community composition from that which occurs under natural conditions. We were able to include five of the seven bat species of our region in our analyses: the little brown bat (*Myotis lucifugus*), big brown bat (*Eptesicus fuscus*), eastern red bat (*Lasiurus borealis*), hoary bat (*L. cinereus*), and silver‐haired bat (*Lasionycteris noctivagans*). On the basis of these species' land cover associations, flight behavior (slow‐ vs. fast‐flying), published observations of foraging around street lights, and known relationships of closely related species with ALAN, we predicted the lighting in our experiment would have an attractive effect on big brown bats, eastern red bats, and hoary bats, and a displacement effect on little brown bats and silver‐haired bats. We expected this would, in turn, significantly change foraging community composition in the presence of ALAN from that which is found in relative darkness.

## 2 MATERIALS AND METHODS

### 2.1 Study site

We conducted our experiment at Great Hollow Nature Preserve in New Fairfield, Connecticut, USA. The preserve is approximately 335 ha and consists of predominantly second‐growth hardwood and mixed hardwood forest. It is contiguous or nearly contiguous with approximately 1,330 ha of additional protected forested lands in Connecticut and neighboring New York State. Development in the surrounding landscape is low density and residential, and the roads bordering the preserve lack street lighting. Our study site is therefore free of any direct, chronic sources of ALAN.

A freshwater stream that runs through the preserve is frequently impounded by American beavers (*Castor canadensis*), which maintains an approximately 4.8 ha area of open and emergent wetland in which we conducted our experiment (41.502260, −73.531317). The wetland is bordered on its eastern and western sides by mature hardwood forest, and on its northern and southern sides by a mix of wet meadow, old field, and shrubland. This area provides open water, riparian, and woodland‐edge habitats that are among the habitat types used for foraging by all species of bats in the region except perhaps the northern long‐eared bat (*M. septentrionalis*), which prefers interior forest (Harvey et al., 2011; Lacki et al., 2007).

### 2.2 Experimental treatments

We conducted our experiment on 65 nights from 20 July to 17 August 2016 and 9 July to 15 August 2017. We randomly assigned each night to one of three treatments: light, dark, or control. On light nights, we operated three 55‐W, 4,400‐lumen, LED utility floodlights (Keystone LED Lighting, Erie, CO, USA) that were mounted on 5‐m tall metal poles and linearly spaced 10 m apart from each other along the eastern edge of the wetland where it transitions to wet meadow and shrubland. The lights were white and had a bimodal spectral pattern typical of LED, with peaks at 450 nm and 590 nm (data provided by manufacturer). We chose to use LED because it is increasingly replacing traditional outdoor lighting as a more energy‐efficient alternative in many cities and towns across North America. The lights had a beam width of 120° and were angled 45° downwards with respect to horizontal, facing west over the wetland. They were turned on a minimum of 3 hr before sunset and remained on for at least 3 hr after sunset. The lights were silently powered by a connection to the preserve's administrative office building approximately 115 m away. On dark nights, the infrastructure was left in place but the lights were off, and on control nights, the lights and poles were taken down to avoid any potential influence of the lighting infrastructure itself on bat activity.

Beginning at sunset each night, we recorded bat activity in the area for 3 hr using a SM4BAT acoustic recorder and SMX‐II microphone (Wildlife Acoustics Inc., Maynard, MA, USA) that was set along the eastern edge of the wetland, 3 m to the north of the center light. The microphone was mounted on top of a 3‐m tall metal pole and angled 45° upwards with respect to horizontal (Britzke et al., 2010; Weller & Zabel, 2002). The microphone had a clear detection cone without any obstruction by vegetation or other objects. The recorder was configured to collect 8‐s, full‐spectrum, triggered sound files at a sampling rate of 384 kHz and gain of 48 dB, following manufacturer recommendations. Sound files were identified to species using the Bats of Connecticut automated classifier in Kaleidoscope Pro 4.0.0 (Wildlife Acoustics Inc., Maynard, MA, USA) set to neutral. Presence of a given species on a given night was accepted when maximum likelihood probability values generated by the software were <0.05 (USFWS, 2020). Because of the similarity of calls among *Myotis* spp. in the northeastern United States and the conservation significance of these species, any automated identifications of little brown bat, northern long‐eared bat, Indiana bat (*M. sodalis*), or eastern small‐footed bat (*M. leibii*) were manually assessed by one of us (AMA) and reclassified as necessary. The manual assessment of these recordings was to confirm whether they were those of a *Myotis* species, and if so, classify them as either little brown bat or northern‐long eared bat based on call duration, call frequencies, and frequency of most energy (Mills et al., 2013). The Indiana bat and eastern small‐footed bat are not known to occur in the county in which we conducted our study (CTDEEP, 2012; Hammerson, 2004) and we therefore discounted the possibility of these species being among our recordings.

### 2.3 Study species

We focused on little brown bat, big brown bat, eastern red bat, hoary bat, and silver‐haired bat because they provided the most sufficient sample sizes for analyses. We detected the two other species in our study area, northern long‐eared bat and tri‐colored bat (*Perimyotis subflavus*), on only two nights and therefore had insufficient data to include them in the study. Big brown bat and little brown bat are year‐round residents in Connecticut and hibernate during the winter in caves or mines, tunnels, buildings, or other artificial structures. They occur during the summer breeding season in a variety of wooded and open habitats across rural, suburban, and urban landscapes. Northeastern populations of the little brown bat declined by more than 90% after the outbreak of white‐nose syndrome (WNS) that began in New York State in 2006 (Turner et al., 2011). Many were projected to become extirpated (Frick et al., 2010) but are now showing signs of stabilization or minor recovery in some areas (Langwig et al., 2017). The species has recently been listed as threatened or endangered in several U.S. States, including Connecticut, and at the federal level in Canada in response to the declines caused by WNS. Big brown bat populations initially declined as a result of WNS, but less precipitously than those of the little brown bat and other myotid bats in the northeastern United States (Turner et al., 2011) and now may be increasing possibly as a result of competitive release (Mayberry et al., 2020; Morningstar et al., 2019) and/or greater resistance to infection (Frank et al., 2014). Eastern red bat, silver‐haired bat, and hoary bat are long‐distance migrants that are sympatric with big brown bats and little brown bats in the northeastern United States during the breeding season and then overwinter in the southern United States Their populations have not been impacted by WNS but face threats from wind energy development (Frick et al., 2017). All five of our study species are nocturnal, aerial insectivores. Based on their wing morphologies, eastern red bat and hoary bat are considered to be fast‐flying species while the little brown bat, big brown bat, and silver‐haired bat are considered to be slow‐flying (Norberg & Rayner, 1987).

### 2.4 Statistical analyses

We conducted statistical analyses in R 3.6.2 and PAST 3.13 and accepted significance in all tests when *p* < .05. We used two‐tailed Fisher's exact tests to compare the number of nights under each lighting treatment that a species was present or absent among our recordings. To compare bat activity among lighting treatments, we first converted the number of sound files identified as a given species on a given night to an activity index that represented the number of 1‐min segments of the recording period that the species was present. This method reduces bias that can be introduced by a small number of highly active individuals (Miller, 2001). We then tested the effects of lighting treatment, Julian date, and year on the activity of big brown bats, eastern red bats, and hoary bats using quasi‐Poisson (log link) generalized linear models (GLM) to account for overdispersion in the counts of those species (Beckerman et al., 2017; Zeileis et al., 2008). For little brown bat and silver‐haired bat, we tested the effects of lighting treatment, Julian date, and year on activity using zero‐inflated negative binomial (ZINB) models (pcsl package; Zeileis et al., 2008) because of an abundance of zeroes and overdispersion in the nonzero count data (Zuur et al., 2009). ZINB models are two‐part mixture models that consist of a logistic regression of presence/absence (i.e., counts of 0 vs. ≥1) to model the probability that a zero value is observed, and a negative binomial regression of counts adjusted for the abundance of zeroes (Hilbe, 2012; Zuur et al., 2009). Temperature and wind speed at the start of each recording period, which were obtained from the nearby (8 km southeast) Danbury Municipal Airport KDXR weather station, did not differ among the three lighting treatments (temperature: *F*_2,53_ = 1.080, *p* = .346; wind: *F*_2, 53_ = 0.209, *p* = .812) and were therefore left out of all analyses in the interest of model simplification (Stone et al., 2012). We used nightly precipitation data from this weather station to limit all analyses to nights when there was no precipitation at least 1 hr before, during, or 1 hr after the 3‐hr recording period.

We compared full models to nested models that had date or year removed using *F*‐tests for the quasi‐Poisson GLMs (Zuur et al., 2009) and likelihood ratio tests (lmtest package) for the ZINB models (Zeileis et al., 2008; Zuur et al., 2009) to determine whether either of these potential covariates could be dropped. For ZINB models, this included removing date and year in turn from the logistic and count models for comparison to their full models (Zuur et al., 2009). We retained date and year for inclusion in a species' final model along with treatment when tests of the full model against the nested model(s) from which they were removed were significant. Because our lighting treatment is a categorical variable with three levels, final ZINB model coefficients provided information about little brown bat and silver‐haired bat activity under light and dark treatments compared only to the control treatment and not to each other. We used the emmeans package (Lenth et al., 2018) to make all pairwise, post hoc comparisons of treatments for the other three focal species whenever there was a significant overall effect of treatment on activity.

We examined whether lighting altered foraging community composition using permutational multivariate analysis of variance (PERMANOVA) on the Bray–Curtis dissimilarity matrix of square root‐transformed activity index data. We chose this method of comparing species assemblages because of its robustness to an abundance of zeroes (Anderson, 2001, 2017). We then used analysis of similarity percentage (SIMPER) to measure the contribution of each species to any observed differences in community composition between pairs of treatments.

## 3 RESULTS

**FIGURE 1:**
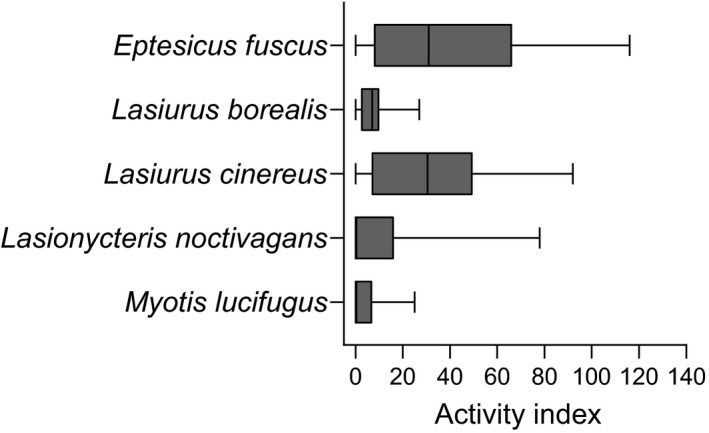
Activity levels of five bat species at Great Hollow Nature Preserve, New Fairfield, Connecticut, USA, 2016 and 2017 (combined across the three lighting treatments). Boxes show the median and 25th to 75th percentiles, and whiskers represent minimum and maximum values

We obtained recordings during clear weather (i.e., no precipitation) on a total of 22, 23, and 13 nights under the light, dark, and control treatments, respectively. Hoary bat was detected on the most nights overall, followed by big brown bat, eastern red bat, little brown bat, and silver‐haired bat (Table 1). Big brown bat was the most active species while little brown bat was the least active (Figure 1).

**TABLE 1:** Percentage of nights bats were detected under light (*N* = 22), dark (*N* = 23), and control (*N* = 13) treatments at Great Hollow Nature Preserve, New Fairfield, Connecticut, USA, 2016 and 2017

Species	Light	Dark	Control	All
*Eptesicus fuscus*	68	96	85	83
*Lasiurus borealis*	82	74	69	82
*Lasiurus cinereus*	91	87	77	86
*Lasionycteris noctivagans*	46	22	23	31
*Myotis lucifugus*	14	65	69	47

### 3.1 Effect of lighting on presence

Big brown bats were present on significantly fewer light than dark nights (*p* = .022), while there was no difference between light and control nights (*p* = .431) or dark and control nights (*p* = .539). Presence of eastern red bats and hoary bats did not differ between any pairs of treatments (all *p* > .340). Silver‐haired bats were present 46% of the time under the light treatment and only 22% and 23% of the time under the dark and control treatments, respectively, but the differences were not significant (all *p* > .12). In the logistic component of the ZINB model, light and dark treatments did not affect the probability of detecting zero silver‐haired bats relative to the control (light: *Z* = −1.301, *p* = .193; dark: *Z* = 0.092, *p* = .926), after dropping year (*p* = .070) and date (*p* = .484). Little brown bat showed the greatest difference in presence between light and dark conditions, with detections on 65% of dark nights and 69% of control nights, but only 14% of light nights. This difference was significant between light and dark treatments (*p* < .001) and light and control treatments (*p* = .002), but not between dark and control treatments (*p* > .999). There was also a greater probability of detecting zero little brown bats on nights when the lights were on than on control nights (*Z* = 3.121, *p* = .002), while there was no such difference between dark and control nights (*Z* = 0.246, *p* = .806).

### 3.2 Effect of lighting on activity

**FIGURE 2:**
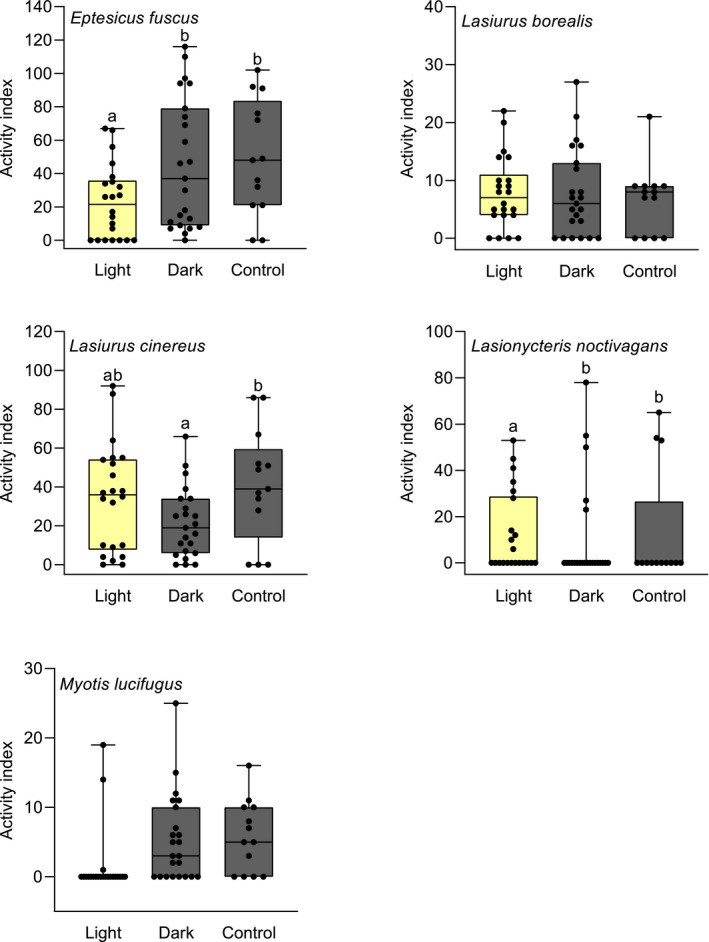
Activity levels of bats under light, dark, and control treatments at Great Hollow Nature Preserve, New Fairfield, Connecticut, USA, 2016 and 2017. Boxes show the median and 25th to 75th percentiles, whiskers represent minimum and maximum values, and black circles represent individual data points. Letters above whiskers indicate significant differences between treatments

Activity of big brown bats averaged only half or less than half on light nights as on dark and control nights (Table 2). This difference in activity among treatments was significant (*F*_2_ = 3.891, *p* = .027) after dropping date (*p* = .146) and year (*p* > .999) and was driven by the lower levels of big brown bat activity on light nights than dark (*Z* = 2.347, *p* = .049) and control (*Z* = 2.403, *p* = .043) nights. Big brown bat activity did not differ between dark and control nights (*Z* = 0.309, *p* = .949; Figure 2). There was no difference in eastern red bat activity among treatments (*F*_2_ = 0.138, *p* = .872; Figure 2) after date (*p* = .870) and year (*p* = .487) were removed. Hoary bat activity declined with date (*F*_1_ = 10.980, *p* = .002), was greater in 2017 than 2016 (*F*_1_ = 7.41, *p* = .009), and differed among treatments (*F*_2_ = 3.959, *p* = .025; Figure 2). Hoary bat activity was not significantly different between light and dark nights (*Z* = −2.250, *p* = .063), or light and control nights (*Z* = 0.554, *p* = .845). Hoary bats were significantly more active on control than dark nights (*Z* = 2.591, *p* = .026; Figure 2).

**TABLE 2:** Species contributions (%) to differences in bat community composition between light and dark nights, and light and control nights at Great Hollow Nature Preserve, New Fairfield, Connecticut, USA, 2016 and 2017

Species	Light versus dark (%)	Light versus control (%)	Light activity index	Dark activity index	Control activity index
*Eptesicus fuscus*	37.6	35.6	22.8 ± 22.1	45.4 ± 38.7	49.2 ± 34.8
*Lasiurus cinereus*	30.0	33.5	34.5 ± 27.5	21.3 ± 17.8	40.7 ± 29.3
*Lasionycteris noctivagans*	16.1	16.8	12.5 ± 17.6	10.1 ± 21.9	13.2 ± 25.3
*Lasiurus borealis*	10.0	7.3	8.0 ± 6.2	7.7 ± 7.5	6.7 ± 5.8
*Myotis lucifugus*	6.3	6.7	1.6 ± 4.9	5.4 ± 6.3	5.8 ± 5.1

Note

Activity index values are means ± *SD*.

Silver‐haired bats were less active on light than control nights (*Z* = −2.082, *p* = .004) while showing no difference between dark and control nights (*Z* = 0.888, *p* = .375; Figure 2) after dropping year (χ^2^ = 0.391, *p* = .532) and controlling for a negative effect of date (χ^2^ = 4.391, *p* = .036; Figure 2). Little brown bat activity also declined with date (χ^2^ = 4.698, *p* = .030) and was unrelated to year (χ^2^ = 0.769, *p* = .381). There was no difference in little brown bat activity between control and dark (*Z* = −0.365, *p* = .715) or light (*Z* = 1.148, *p* = .251; Figure 2) treatments even though little brown bats were rarely present on light nights. This was due to high counts of little brown bats that occurred on two of the only three nights on which they were detected while the lights were on. With these two outliers omitted, there was significantly lower little brown bat activity on light than control nights (*Z* = −4.952, *p* < .001) and no difference in activity between dark and control nights (*Z* = −0.336, *p* = .737; Figure 2).

### 3.3 Effect of lighting on species composition

Species composition was different among treatments (*F* = 2.800, *p* = .002). This was due to dissimilarities between the light treatment and both the dark (*F* = 3.663, *p* = .003) and control (*F* = 2.989, *p* = .016) treatments. Species composition did not differ between the dark and control treatments (*F* = 1.388, *p* = .228). Reduced activity of big brown bats along with no change in hoary bat activity when the lights were on contributed the most to the differences in community composition between light and dark conditions (Table 2). Hoary bat replaced big brown bat as the dominant species in the community when the lights were on compared with the other treatments (Table 2). Little brown bat showed the greatest relative difference in activity between light and dark conditions, nearly being removed from the foraging community by the lighting (Tables 1 and 2), but its rarity and much lower overall activity than other species reduced its contribution to the dissimilarities in species composition (Table 2).

## 4 DISCUSSION

As concerns about the impacts of light pollution to biodiversity have increased around the world, its effects on North American bats have remained understudied and largely unknown. We experimentally tested the effects of ALAN on a community of free‐living bats in the northeastern United States to document these species' changes in foraging activity in response to the acute introduction of LED lighting to an otherwise dark environment. We observed clear light aversion by the two nonmigratory species of bats while finding neutral or mixed evidence for light avoidance or attraction among the three species of migratory tree bats. This resulted in a significant change in foraging community composition in the presence of ALAN. Responses to the lighting were consistent with our predictions for some species based on previous lighting studies, their associations with light‐polluted landscapes, and/or their flight behavior, while for other species they were not. The study species that exhibited the strongest aversion to the lighting treatment, the little brown bat, is also currently the one of greatest conservation need, highlighting the importance of considering and mitigating impacts of ALAN to this imperiled species as managers attempt to recover its eastern populations from steep declines.

We expected to observe a decrease in little brown bat activity in response to our lighting treatment because European congeners have been widely found to be light‐averse (Rowse et al., 2016) and the few studies of the effects of ALAN on little brown bats and other North American myotid bats also point toward a negative effect (Bradbury & Nottebohm, 1969; Cravens & Boyles, 2019; McGuire & Fenton, 2010). Cravens and Boyles (2019) recently provided the most direct evidence for light aversion by little brown bats by showing significantly lower foraging activity in experimentally illuminated sites than dark sites in Missouri, USA. In Canada, little brown bats have been reported to feed on insects around streetlights (Acharya & Fenton, 1999; Fenton et al., 1983), but with a preference for foraging in darker areas (Furlonger et al., 1987). McGuire and Fenton (2010) noted that an external light on a recreational trailer deterred little brown bats while also impeding their orientation and obstacle avoidance abilities, although distress calls from other little brown bats may have contributed to the effect. Captive studies of little brown bats have similarly found artificial lighting to negatively affect foraging behavior (Alsheimer, 2011) and obstacle avoidance (Bradbury & Nottebohm, 1969). Little brown bats in our study seldom occurred in the site when the lights were on relative to when conditions were dark. The lighting treatment positively affected the probability of counting zero little brown bats or negatively affected little brown bat activity, depending on whether outliers were removed, but in either case, artificial illumination of the wetland caused little brown bats to forage there less than under natural conditions. Collectively, our observations along with those of others provide strong evidence that the little brown bat is a light‐averse species that may experience restricted foraging habitat availability and competitive disadvantages against other bats in light‐polluted environments.

As with the little brown bat, but counter to our predictions, we observed the experimental lighting of our study site to negatively affect big brown bats. They were present over the wetland on fewer nights and were less active when the lights were on than they were during dark conditions. Even though they are slow‐flying, we expected the opposite to occur because of the ubiquity of big brown bats in metropolitan areas where ALAN is pervasive (e.g., Loeb et al., 2009; Schimpp et al., 2018) and observations of big brown bats and European congeners being attracted to insect concentrations around street lights (Geggie & Fenton, 1985; Furlonger et al., 1987; Rydell, 1991, 1992; Catto et al., 1996; but see Azam et al., 2018). However, an experiment similar to ours also found big brown bats to avoid naturally dark habitats that were temporarily treated with acute sources of ALAN (Cravens & Boyles, 2019). A major difference between these observational and experimental studies is the spectral composition of the lighting. Observational studies of big brown bats (Furlonger et al., 1987; Geggie & Fenton, 1985) and European congeners (Catto et al., 1996; Rydell, 1991, 1992) around street lights have involved older lighting technologies such as mercury or sodium vapor, while both experimental studies of big brown bats (Cravens & Boyles, 2019, this study) used LED. Compared with older lighting technologies, LED can attract a different taxonomic composition of insects and fewer insects overall (van Grunsven et al., 2014). In turn, light‐tolerant bat species that commonly exploit the concentration of insects around mercury or sodium vapor lights are not always attracted to LED lights (Lewanzik & Voigt, 2017). However, we and Cravens and Boyles (2019) not only found that big brown bats were not attracted to the LED lighting (i.e., presence and activity were not greater during lighting treatments than under control conditions), but that they actively avoided it (i.e., presence and activity were lower during lighting treatments than under control conditions). Therefore, differences in spectral composition and prey densities alone cannot explain why a species that is attracted to mercury and sodium vapor street lights and commonly inhabits chronically light‐polluted landscapes was displaced by the LED lighting in these two experimental studies. Rather, we suspect there to be habituation, phenotypic plasticity, and/or selection for light tolerance in big brown bats living in environments with long‐term, chronic sources of ALAN, such as street lights and many light sources in heavily developed landscapes, while those in darker areas that are relatively naive to ALAN negatively react to sudden, acute lighting of their environment (Russo et al., 2019). Long‐term studies are needed to determine whether such processes indeed occur in big brown bats and other bat species, allowing them to eventually habituate to light pollution.

Eastern red bats and hoary bats were not present more often or more active when the lights were on than they were under dark conditions, which was also contrary to our expectations. Eastern red bats are common to light‐polluted, urban areas (e.g., Parkins & Clark, 2015; Schimpp et al., 2018) and in darker landscapes, are often seen feeding around lights that concentrate insect prey (Furlonger et al., 1987; Hickey et al., 1996). As early as 1969, they were described in the literature as being exploitative of the attraction of insects to lights possibly more than any other bat (Barbour & Davis, 1969). Along with these anecdotes, quantitative comparisons of eastern red bat activity between lit and dark sites in Ontario, Canada (Furlonger et al., 1987) and Missouri, USA (Cravens & Boyles, 2019) have demonstrated a clear preference by this species for foraging in spaces artificially illuminated with either LED (Cravens & Boyles, 2019) or older lighting technologies (Furlonger et al., 1987) over relatively dark areas. Hoary bats have similarly been shown to be attracted to, and significantly more active around, artificial lighting (Acharya & Fenton, 1999; Fenton et al., 1983; Furlonger et al., 1987). The closely related Hawaiian hoary bat (*L. semotus*) is also known to regularly feed around lights (Belwood & Fullard, 1984; Jacobs, 1999). Hickey et al. (1996) described hoary and eastern red bats foraging together around mercury vapor street lights on a nightly basis, overlapping with each other in time and diet. Eastern red bats and hoary bats are adept at pursuing prey at relatively fast flight speeds and in open habitats, which are characteristics that are common among light‐tolerant bat species (Rowse et al., 2016). While we did not observe a significant attractive effect of our lighting treatment on these species, there was no displacement effect either, further supporting the categorization of eastern red and hoary bats as light‐tolerant species.

We are not aware of any previous studies of the effects of ALAN on silver‐haired bats. However, silver‐haired bats are slow flyers and feed in small clearings (Barclay, 1985; Norberg & Rayner, 1987), which are characteristics that are shared by many light‐averse bat species (Rowse et al., 2016). Also, unlike many other North American bats, there do not appear to be any published observations of silver‐haired bats feeding around artificial light sources. In several national parks in Canada, for example, silver‐haired bats were not among the bats documented feeding around street lights even though they were known to occur elsewhere within those protected areas (Fenton et al., 1983). Some studies have found silver‐haired bats to be negatively associated with urban land cover in which ALAN is pervasive (Dixon, 2012; Li & Wilkins, 2014), but others have found the opposite (Gehrt & Chelsvig, 2004; Schimpp et al., 2018). Our experiment yielded ambiguous results that did not consistently indicate an attraction or displacement effect of lighting on silver‐haired bats. Silver‐haired bats were present on at least two times as many light nights as dark or control nights, and the lighting did not increase the probability of detecting zero silver‐haired bats. However, silver‐haired bat activity was significantly lower under light than dark conditions. We therefore cannot conclusively determine whether the silver‐haired bat is tolerant of, or averse to, LED lighting, and we encourage further work on this species to help clarify its relationship with ALAN.

The aversion to lighting by some of our study species but not others caused a significant change in species composition in the presence of ALAN. This could alter competitive balances from natural conditions, although direct competition for food between light‐tolerant eastern red bats and hoary bats, and light‐averse big brown bats is likely limited because of different prey preferences. Eastern red bats and hoary bats prefer moths (Clare et al., 2009; Hickey et al., 1996), while big brown bats prefer beetles (Clare et al., 2014; Cravens et al., 2018). This may partly explain why there was no increase in eastern red bat or hoary bat activity on light nights in response to the substantial decrease in big brown bat activity. However, even in the absence of interspecific food competition, displacement of light‐averse species from light‐polluted areas will reduce the amount of foraging habitat available to them within the landscape and thereby potentially limit their populations while light‐tolerant species increase in relative abundance within the community. Competitive exclusion by light‐tolerant common pipistrelles (*Pipistrellus pipistrellus*) has been implicated as a cause of decline in the light‐averse lesser horseshoe bat (*Rhinolophus hipposieros*) in Switzerland (Arlettaz et al., 2000) and intermittent lighting around sports stadiums in Germany was found to change the species assemblage of foraging bats relative to dark conditions (Schoeman, 2016), but community‐level impacts of ALAN to bats otherwise remain largely unknown and in need of study (Rowse et al., 2016; Stone et al., 2015).

Many North American bat populations face threats from habitat loss, wind energy development, cave tourism, environmental contaminants, climate change, and most significantly for hibernating species, WNS (Hammerson et al., 2017). Our results indicate that the introduction of only a small degree of ALAN to a habitat within a relatively dark landscape is a potentially significant form of habitat degradation for some North American bats—one that will continue to spread in concert with human population growth and development across the continent. The conditions of our experiment perhaps most closely simulate the light pollution introduced by exurban development, which describes low‐density residential development in rural areas, often near or within protected areas or other lands of high conservation value (Radeloff et al., 2010; Theobald, 2005). Exurban development is the fastest‐growing form of land‐use change in the United States (Brown et al., 2005). Its impacts to wildlife extend well beyond the physical footprint of disturbance and are thought to be driven by homeowner behaviors, including the use of outdoor lighting at night, more so than the structural alteration of the habitat (Glennon & Kretser, 2013; Glennon et al., 2014). As rural areas become more exurban, bats outside cities and suburbs will more frequently encounter ALAN, and light‐averse species will be increasingly challenged to either habituate to the environmental change and compete with light‐tolerant species, or find dark refugia.

The effects of ALAN on declining species that are affected by WNS, some of which are listed as endangered or threatened, have been surprisingly understudied given the strategy of some regulators to minimize other anthropogenic stressors until practical and effective ways of directly managing the disease can be identified or until selection for resistance can occur (e.g., USFWS, 2011, 2015). ALAN has been found to delay emergence time and displace bats from preferred commuting routes to their foraging areas, both of which are expected to have energetic costs that could manifest in reduced reproduction rates and lower fitness; yet, the population‐level consequences of ALANs impacts to bats are still largely unknown (Rowse et al., 2016; Stone et al., 2009, 2015). This lack of information hinders the ability of regulatory agencies in North America to conduct science‐based assessments of potential impacts to bats from proposed developments and other land‐use changes that would introduce new sources of artificial lighting to an area. It is also a barrier to the development of new regulations that are potentially needed to protect North American bats from ALAN encroaching into dark landscapes as land‐use change progresses. Further research on the ways in which, and to what extent, different lighting technologies, colors, and intensities affect these species is therefore urgently needed and should be a priority in conservation planning for North America's bats.

## CONFLICT OF INTEREST

The authors declare that no conflicting interests existed in the completion of this research.

## AUTHOR CONTRIBUTIONS

**Chad L. Seewagen:** Conceptualization (lead); formal analysis (lead); investigation (lead); writing‐original draft (lead); writing‐review & editing (lead). **Amanda M. Adams:** Formal analysis (supporting); investigation (supporting); writing‐original draft (supporting); writing‐review & editing (supporting).

## Data Availability

The data used in this study are available from the Dryad repository (https://doi.org/10.5061/dryad.6hdr7sr0d).
